# Reactive control in suicide ideators and attempters: An examination of the congruency sequence effect in cognitive and emotional Simon tasks

**DOI:** 10.1371/journal.pone.0295041

**Published:** 2023-11-30

**Authors:** Hyejin J. Lee, Joohyang Kang, Hwajeong Yu, Chae Eun Lim, EunByeol Oh, Jong Moon Choi, Sungeun You, Yang Seok Cho

**Affiliations:** 1 School of Psychology, Korea University, Seoul, Republic of Korea; 2 Department of Psychology, Chungbuk National University, Cheongju, Chungbuk, Republic of Korea; Edge Hill University, UNITED KINGDOM

## Abstract

Reactive control is the cognitive ability to adjust thoughts and behaviors when encountering conflict. We investigated how this ability to manage conflict and stress distinguishes suicidal from nonsuicidal individuals. The hypothesis was that suicidal individuals would show poorer reactive control when faced with conflict generated by emotional than neutral stimuli. Hence, individuals with a lifetime history of suicide ideation or attempt and nonsuicidal controls were tested in cognitive and emotional Simon tasks. We examined the congruency sequence effect (CSE) in the Simon tasks as an indication of the efficiency of reactive control in resolving conflict. Whereas controls demonstrated significant CSEs in both tasks, suicide attempters showed a significant CSE in the cognitive task but not in the emotional task. Suicide ideators, on the other hand, displayed marginally significant CSEs in both tasks. Comparing groups with pairwise comparison demonstrated that the difference in CSE was significant only in the emotional task between attempters and controls. Our findings of attempters’ inefficiency in adjusting reactive control during the emotional task reflect cognitive inflexibility in coping with conflicting situations during which suicidal individuals become vulnerable to suicide attempts in states of negative emotion.

## Introduction

Suicide is an outcome of various factors interacting [1, 2], making the study of causes and conditions of suicide highly challenging. Psychological models of suicidal behavior have suggested various risk factors for suicidal behavior, and an important issue raised is which factor distinguishes people who enact suicide from those who only think of it [3–5]. One of the aspects relatively less investigated but may play a critical role in enacting suicide is the cognitive aspect: How may suicide attempters differ in cognitive processing, especially in stressful situations of feeling urges to commit suicide? Most suicidal acts are reactive to internal or external stress [6–8]. According to the cognitive model of suicidal behavior, cognitive processes in high stress prompt suicidal acts [8]. Particularly, cognitive inflexibility may increase the risk of committing suicide through deficits in problem-solving [9, 10]. Suicidal individuals generate fewer alternatives in solving problems and lack controllability under challenging situations than nonsuicidal individuals [11]. Accordingly, failing to efficiently react to stress by regulating thoughts and actions may be one critical cognitive factor of why people may enact suicidal behavior.

To manage stress, one can focus on goal achievement rather than on intrusive thoughts or stress-inducing events. However, people become susceptible to distractions when successful regulation to selectively focus on goals is disturbed [12–14]. Stroop tasks require responding to task-relevant information (e.g., the color of the word in a color-word Stroop task) while ignoring task-irrelevant information (e.g., the word meaning in a color-word Stroop task). Failing to inhibit task-irrelevant information results in longer response time (RT) and higher percent error, which is referred to as the congruency effect [14]. When suicidal individuals were tested in Stroop tasks and compared with nonsuicidal individuals, they showed larger congruency effect resulting from being more disturbed by distractions [15–19]. Importantly, human cognition can actively adapt to stress and conflict instigated by distractions [20–22]; when conflict is detected due to mismatches between task-relevant and task-irrelevant information, reactive control resolves the conflict by transiently reactivating goals and inhibiting task-irrelevant information [22, 23]. As a result, one becomes less interfered by distractions after experiencing conflict. Accordingly, reactive control facilitates subsequent task performance and enables one to proceed toward goals even during conflicting situations.

The efficiency of reactive control is indexed by the congruency sequence effect (CSE) or Gratton effect of congruency tasks (e.g., Stroop task, Simon task) [24]. In Simon tasks, a target appears on the left or right of central fixation on a computer monitor, and individuals respond to a nonspatial stimulus feature of the target, such as its color. Despite the irrelevance to the task of responding to the feature, the target location induces conflict and impairs task performance when it is incongruent rather than congruent to the location of the correct response key associated with the feature [25, 26]. That is, people respond more slowly or inaccurately when the target appears on the opposite side of the response key. This performance impairment resulting from the conflict between task-irrelevant and task-relevant dimensions in Simon tasks is also indicated as the congruency effect and is calculated as the difference in RT or percent error between incongruent and congruent trials. Interestingly, the congruency effect is affected by whether a previous trial was incongruent or congruent [27]. If a previous trial was incongruent, reactive control facilitates the performance of the subsequent trial with its inhibition of task-irrelevant information [28]. As a result, the size of the congruency effect is significantly reduced. This phenomenon of the congruency effect decreasing after experiencing conflict is the CSE. It reflects the active adjustment of reactive control in coping with conflict.

Previous studies using congruency tasks have mostly focused on comparing the size of the congruency effect and its indication of interference control between suicidal and nonsuicidal individuals [15, 16, 18, 19]. Those studies have examined if suicidal individuals are more susceptible to distracting information or display more difficulty selectively focusing on task-relevant information compared to nonsuicidal individuals. In contrast, how interference control can be dynamically adjusted by reactive control processes with the CSE has been less examined. Assessing the ability of suicidal individuals to reactively control interfering stimuli in a conflict-induced task would test if suicidal individuals may experience more difficulty adapting to interference in conflicting situations compared to nonsuicidal individuals.

Therefore, to investigate if reactive control can distinguish suicidal and nonsuicidal individuals, we examined the adaptation of reactive control using Simon tasks. Among congruency tasks, the control to resolve conflict instigated in Simon tasks is exerted on inhibiting task-irrelevant information rather than on facilitating task-relevant representations [28]. Accordingly, by comparing the CSE in Simon tasks between suicidal and nonsuicidal groups, we aimed to investigate how actively inhibiting distractors relates to suicidal behaviors.

Previous research suggested that the absence of CSE in a particular group indicates cognitive inflexibility (e.g., [29]). Cognitive flexibility is a construct that encompasses various cognitive functions [30, 31]. However, we focused on the ability to adapt behaviors to changing situations [30]. Suicidal individuals may experience less efficiency in adapting their cognitive processing than nonsuicidal individuals in certain circumstances [8]. Failing to flexibly modify thoughts and behaviors to resolve conflict that interferes with goal achievement can lead to frequent disturbance by distractions. Accordingly, we hypothesized that reactive control may explain how cognitive processing differs in suicidal individuals from nonsuicidal individuals; we assumed that poorer adaption of reactive control may increase one’s vulnerability to intrusive thoughts or rumination related to suicide [32–34]. We expected that this group difference would be demonstrated by a significant group difference in CSE, due to a significantly smaller CSE in suicidal compared to nonsuicidal individuals.

A crucial question was in which circumstances suicidal individuals would experience inefficient reactive control. Considering that negative life events and psychosocial stress are closely associated with suicide attempts [6, 35–37], we hypothesized that suicidal individuals would experience poor control in conflicting situations when the conflict is caused by emotional stimuli, such as human faces. We considered the emotional context of when regulatory functions are implemented to be critical in explaining suicidal behaviors because previous research demonstrated that the difference between suicidal and nonsuicidal groups depended on emotion [38, 39]. For example, You et al. [39] showed that compared to nonsuicidal controls, individuals with a lifetime history of suicide ideation or attempt were poorer in response inhibition in a threat context but not in a non-threat context. Their findings demonstrated that emotion plays a key role in distinguishing between suicidal and nonsuicidal individuals regarding their ability to regulate behaviors. However, this study did not directly compare cognitive and emotional tasks in explaining how negative emotion affects suicidal behaviors.

Hence, here we tested the hypothesis that suicidal individuals would show poorer reactive control during a task that displays negative emotional stimuli. We compared the performance of suicidal and nonsuicidal individuals in a cognitive Simon task and an emotional Simon task. The cognitive task was to respond to the color of a square, and the emotional task was to respond to the biological sex of a face whose expressions were sad for half of the blocks. Responding to the biological sex instead of the facial expression makes task-relevant representations of the face non-emotional and task-irrelevant representations emotional. This design intends that the conflict is instigated by task-irrelevant emotional information, assuming that suicidal behaviors are related to difficulty in dealing with distracting information in negative emotional states. The two tasks were performed alternatively on a trial-by-trial basis to control for confounds in CSE (see Methods for details). The prediction was that if suicidal individuals are impaired in adjusting reactive control by the conflict generated during the processing of human faces rather than simple geometric shapes, suicidal individuals would show a significantly smaller CSE during the emotional task compared to the cognitive task. Furthermore, if the impairment is particularly due to negative emotion displayed by the faces, the CSE would be reduced in sad blocks of the emotional task compared to neutral blocks.

We divided our sample into three groups based on clinical interviews that assessed suicidal behaviors: Suicide ideators, suicide attempters, and nonsuicidal controls. Distinguishing individuals who have had suicidal thoughts but did not act on them from those who had taken actions to execute the thoughts were based on the ideation-to-action framework [3–5]. The framework suggests that suicidal behaviors exist on a continuum from ideation to action and cognitive control acts as a moderating factor that facilitates the progression from ideation to attempt. Our goal was to investigate if the efficiency of reactive control can distinguish attempters from nonsuicidal controls as well as ideators. The prediction was that if ideators would display more efficient reactive control than attempters because they do not execute suicidal thoughts, attempters would perform the poorest reactive control among the three groups.

During the clinical interviews, cognitive flexibility and current depressive symptoms were assessed using self-report measures [30, 40]. We used the cognitive flexibility measure to investigate if its subscale of measuring the ability to perceive difficult situations as controllable would be positively correlated with participants’ actual performance in controlling conflict. The current depressive severity measure was used as a covariate to examine if differences in depression severity alternatively explain the hypothesized group effects in the CSE. If the suicidal groups differ from controls in the efficiency of adapting reactive control even when covarying for the depressive symptom severity, the results exclude the possibility that depressive symptoms contribute to the group effect instead of lifetime suicidal behaviors.

## Method

### Participants

The target sample size was 40 per group. We decided on this number with a power calculation using the open-source R package, BUCSS [41]. We used its function for mixed analyses of variance (ANOVAs) to examine possible group differences in CSE. As the function requires inputting the observed F-value and the total sample size of a previous related study, we selected the numbers from You et al.’s [39] study, which also examined differences in a cognitive control function across three groups differing in suicidal behaviors. With inputting the F-value of 6.02 and the total sample size of 122, the necessary per-group sample size to achieve a power of *1-β* = .75 at an *α* = .05 was 42.

We recruited community adults with a lifetime history of suicide ideation or attempt in two cities in South Korea, Cheongju and Seoul, using flyers and online postings. We placed the flyers at local hospitals and uploaded online postings on university student community websites. Nonsuicidal community controls were recruited using the same strategy. Among a total of 163 adults, we excluded 26 who reported a history of nonsuicidal self-injury (NSSI) without a history of suicidal thoughts or attempts. We divided the remaining 137 participants (80 females 57 males; age *M* = 22.82, *SD* = 3.03) into three groups based on the clinical interviews, resulting in 57 with a history of suicide attempt, 40 with a history of suicide ideation but no attempt, and 40 with no history of suicide ideation and attempt. The majority of participants identified themselves as college students (74.45%) or college graduates (13.87%). Monthly income ranged from less than one million KRW (53.28%) to more than five million KRW (16.79%).

Informed consent was obtained from all participants. The study was approved by the institutional review boards of Chungbuk National University (CBNU-201804-SBETC-615-01) and Korea University (KU-IRB-17-17-A-1).

### Assessment of suicidal behavior

We used the Columbia-Suicide Severity Rating Scale to assess suicidal behaviors during the clinical interviews (C-SSRS, [42, 43]). We used the version that is translated into Korean [44]. All interviewers had completed the online training provided by the original developer of the C-SSRS. They also participated in rehearsals and observation sessions led by a doctoral-level clinical psychologist before performing actual interviews. All interviews were supervised by a doctoral-level clinical psychologist.

Based on the interviews, participants were divided into ideators, attempters, and controls. Participants, who reported a history of suicidal ideation but no attempt, were classified as ideators. This ideation group encompassed participants with at least one positive response to the question numbers two to five of the C-SSRS severity questions. Participants, who reported a history of suicide attempt, were classified as attempters. Attempters were subdivided into actual, interrupted, and aborted attempters as the C-SSRS further specifies suicidal behaviors. However, we did not use this subdivision in running data analysis because previous findings indicated that actual, interrupted, and aborted attempters do not significantly differ but instead share similar suicide risk characteristics [45, 46]. An interrupted attempt is an action executed with an intent to end one’s life but stopped by someone or something external to the attempters. An aborted attempt is also executed with an intent to die but stopped by the attempters themselves. We considered interrupted and aborted attempts also as suicide attempts according to the broadly accepted definition of a suicide attempt as a self-directed injurious behavior with the intention to end one’s life [47]. Consistently, prior research demonstrated that individuals reporting interrupted or aborted attempts without a history of actual attempts do not significantly differ from individuals reporting actual attempts as they show similar levels of suicidal desires and acquired capability for suicide [45, 46]. Lastly, participants, who had no lifetime history of suicidal ideation and attempt, were classified as controls. Controls, who reported positively only to the “wish to be dead” question, were not classified into the suicidal groups. We disregarded passive suicidal ideation because the majority of epidemiological studies of suicide has asked about active suicidal ideation when assessing suicidal ideation [48–50], and research on passive ideation is yet unclear.

### Cognitive and emotional Simon tasks

#### Apparatus

Participants were unrestrainedly seated 60 cm away from a 22-inch LCD monitor of 1,024 x 768 pixels and a 60-Hz refresh rate. Responses were collected using a standard computer keyboard. The experiment was programmed with *MATLAB* (www.mathworks.com) and Psychtoolbox 3.0.11.

#### Stimuli

The target stimulus was either a colored square or a face image. The square (approximately 3.33° x 3.33° in visual angle) was either red or yellow. We selected the face images (approximately 8.3° x 10.3°) from the Korea University Facial Expression Collection 2^nd^ edition (KUFEC-II, [51]), a stimulus set of facial expressions displaying neutral and six basic emotions of Korean young adults (age *M* = 24.72). We selected 16 sad faces (eight females and eight males) and 16 neutral faces (eight females and eight males) from the set. We chose sad expressions because depression is strongly associated with suicidal behavior [1].

#### Task procedure

**Fig 1:**
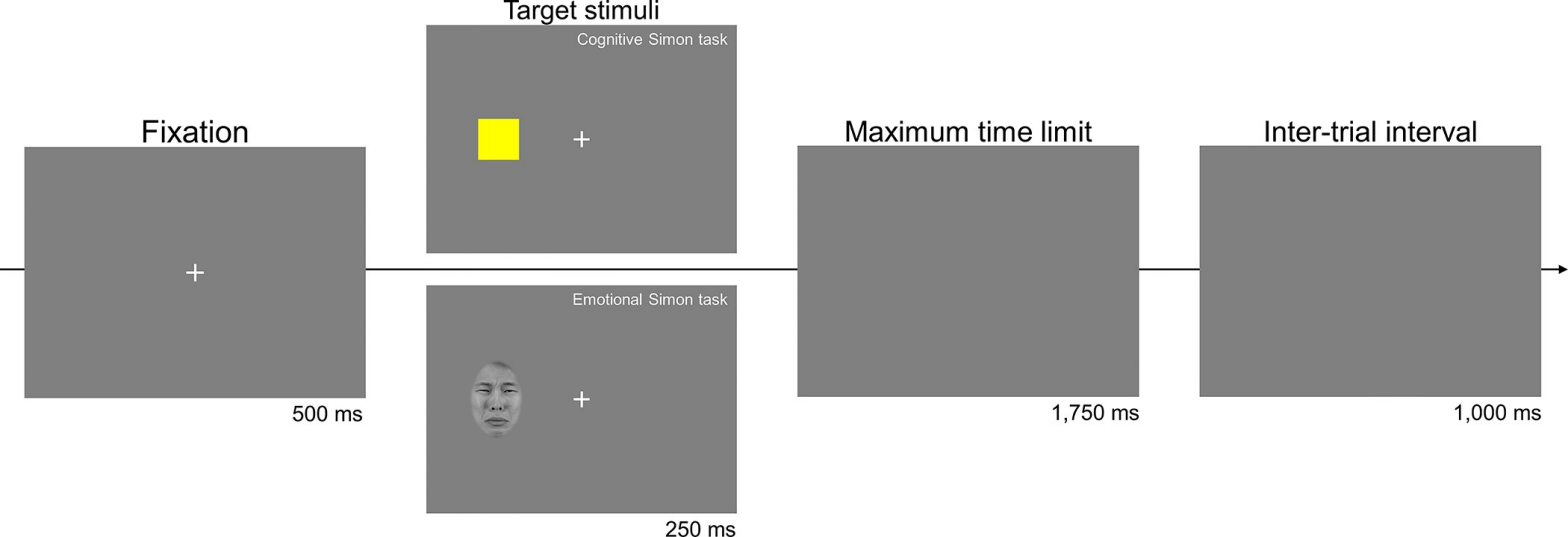
The trial sequence of the Simon tasks. The cognitive Simon task, responding to the color of a square, and the emotional Simon task, responding to the biological sex of a face, were given in turn. The faces displayed sad expressions for half of the blocks and neutral for the other half. A trial was congruent when the location of the target stimulus from the fixation cross and the hand position of the correct response key were aligned on the same side but incongruent when they were in opposite positions. The example face image is from KUFEC-II, an emotional and neutral face image set of Korean young adults and was authorized to be used and published [51].

Experimenters made sure that the midline of a participant’s body was aligned centrally across the midpoint of the monitor. After participants performed a practice block of 34 trials, they performed the main experiment, composed of eight blocks of 66 trials. Each trial started with a white fixation cross appearing for 500 ms in the center of the monitor. Participants performed two tasks: a color Simon task (cognitive task) and a face Simon task (emotional task). Hence, the target stimulus was either a colored square or a face image, depending on the required task. The target appeared either at the left or right of the fixation cross. While our focus was to compare performance between the two Simon tasks, we adopted an experimental design of alternatively performing two tasks on a trial-by-trial basis instead of presenting two tasks in separate blocks. Participants also performed the two tasks in turn with distinguished response keys for each task; participants responded to the color of a square for the cognitive task by pressing the “f” key for red and the “j” key for yellow with their index fingers and responded to the biological sex of a face by pressing the “d” key for males and the “k” key for females with their middle fingers for the emotional task. As a result, the two tasks, which were performed alternatively, had distinguished stimulus and response features. With this design, the CSE observed in our experiment could not be attributed to feature integration or contingency learning, which are bottom-up confounds that could yield the CSE pattern [52]. Lim and Cho [53] demonstrated by presenting two Simon tasks in this design that the CSE can occur without feature integration and contingency learning. A trial was categorized as congruent if the target appeared on the same side of the response location and incongruent if it appeared on the opposite side. The faces displayed sad expressions for half of the blocks and neutral expressions for the other half. The sad and neutral blocks were given in turn with their order counterbalanced across participants. The target stimulus appeared for 250 ms. Participants were required to respond within 2,000 ms from the target onset. Trials were separated with an interval of 1,000 ms (Fig 1).

For each task, trial sequence was generated such that each congruency sequence [cC, cI, iC, iI; the combination of lowercase and uppercase letters indicates current trial congruency (C: congruent, I: incongruent) preceded by either a congruent (c) or an incongruent (i) previous trial] occurred for the same number of times. The CSE was calculated as the difference in congruency effect between trials whose previous trials were congruent and trials whose previous trials were incongruent [(cI-cC)-(iI-iC)]. While the two tasks were distinguished in that they did not share stimulus and response features, the CSE across the two tasks could be calculated as they shared task-irrelevant spatial dimensions [53]. This was addressed in previous studies that two different tasks could yield a cross-task CSE as long as the tasks share the same task-irrelevant stimulus dimension and response dimension [53, 54].

### Self-report measures

#### Cognitive Flexibility Inventory (CFI)

We assessed cognitive flexibility using the CFI [30]. The CFI measures cognitive flexibility in two subscales, the *Alternatives* and *Control*. The Alternatives subscale measures the ability to perceive and generate alternative solutions to difficult situations, and the Control subscale measures the tendency to perceive difficult situations as controllable. The psychometric properties of the two subscales indexed by Cronbach’s alpha ranged from good to excellent [30]. The version translated into Korean also showed consistent reliability levels with Cronbach’s alpha of .86 for the total, .87 for the Alternatives subscale, and .84 for the Control subscale. We used this translated version during the clinical interviews.

#### Patient Health Questionnaire-9 (PHQ-9)

Current depressive symptoms were measured using the PHQ-9 [40]. We used the version that is translated into Korean [55]. Its reliability indexed by Cronbach’s alpha is .95 [55]. Participants were asked how much they have experienced depressive symptoms for the past two weeks on a 4-Likert scale from 0 (none) to 3 (almost every day). We used the sum score as a covariate when conducting statistical tests on Simon task measures.

## Results

### Preliminary analyses

No significant group differences were present in age, *F*(2, 134) = .38, *p* = .68, gender, *χ*^2^(2) = .91, *p* = .63, educational level, *χ*^2^(8) = 12.75, *p* = .12, and income, *χ*^2^(10) = 10.43, *p* = .40. The C-SSRS scores indicated a significant group difference in the suicide ideation severity, *F*(2, 134) = 497.86, *p* < .001, in which attempters scored higher than ideators and controls, and ideators than controls, all *ps* < .001. Furthermore, the PHQ-9 scores indicated a significant group difference, *F*(2, 134) = 497.86, *p* < .001, in which attempters scored higher than ideators, *p* < .05 and controls, *p* < .001 and ideators than controls, *p* < .001. We identified 57 attempters as 45.6% (*n* = 26) multiple attempters, 45.6% (*n* = 26) actual attempters, 22.8% (*n* = 13) interrupted attempters, and 56.1% (*n* = 32) aborted attempters. In addition, 47.4% (*n* = 27) reported histories of NSSI (see Table 1).

**Table 1:** Sociodemographic and clinical characteristics of the sample by group.

	Attempters *(n* = 57)		Ideators *(n* = 40)		Controls *(n* = 40)		*Statistics*	
Variable	*n*	*%*	*n*	*%*	*n*	*%*	*F / χ* ^2^	*p*
Age in years (*M*, *SD*)	23.09	2.99	22.58	3.21	22.70	2.95	*F* = .38	.68
Gender (% Female)	36	63.16	22	55.0	22	55.0	*χ*^2^ = .91	.63
Education							*χ*^2^ = 12.75	.12
High school or below	5	8.77	0	0	0	0		
University attending	35	61.40	35	87.5	32	80		
University graduates	11	19.30	3	7.5	5	12.5		
Graduate school and above	6	10.53	2	5	3	7.5		
Income							*χ*^2^ = 10.43	.40
Under 1 million KRW	27	47.37	21	52.5	25	62.5		
1–2 million KRW	6	10.53	2	5	2	5		
2–3 million KRW	5	8.77	1	2.5	4	10		
3–4 million KRW	5	8.77	6	15	0	0		
4–5 million KRW	5	8.77	3	7.5	2	5		
5 million KRW and over	9	15.79	7	17.5	7	17.5		
C-SSRS								
Suicide ideation								
Wish to be dead	57	100	38	95	10	25		
Active suicidal thoughts	57	100	40	100	0	0		
Active ideation with any method	57	100	34	85.5	0	0		
Active ideation with intent	52	91.2	16	40.0	0	0		
Active ideation with plan and intent	17	29.8	3	7.5	0	0		
Suicide ideation severity (*M*, *SD*)	4.21	.56	3.32	.83	0.25	.44	*F* = 497.86	.00
Suicide attempt subtypes								
Actual	26	45.6						
Interrupted	13	22.8						
Aborted	32	56.1						
Suicide attempt frequency								
Single	31	54.4						
Multiple	26	45.6						
NSSI history	27	47.4	0	0	0	0		
PHQ-9 (*M*, *SD*)	13.14	6.17	9.90	5.78	3.75	3.73	*F* = 35.14	.00

*Note*. C-SSRS = Columbia-Suicide Severity Rating Scale; NSSI = Nonsuicidal Self Injury. PHQ-9 = Patient Health Questionnaire-9.

1 million KRW ≈ 840.15 USD. The percentage of suicide attempt subtypes sums over 100% because our sample includes individuals with multiple attempters.

### Group differences in cognitive and emotional Simon tasks

#### RT data

We measured RT and accuracy. We report ANOVA results separately conducted on RT and percent error [(1-accuracy)*100] data. For preprocessing data, we excluded the first two trials of every block, trials whose mean RTs were three *SDs* away from conditional means, and trials following outliers or incorrect trials. Mean correct RT was calculated for each participant as a function of task (cognitive or emotional tasks), block (sad or neutral blocks), previous trial congruency (congruent or incongruent), and current trial congruency (congruent or incongruent). A mixed ANOVA was conducted with the four factors as within-subject variables and group (ideators, attempters, or controls) as a between-subject variable (see Table 2).

**Table 2:** Mean RTs and percent error (*SD*s in parentheses) as a function of task, block, previous trial congruency (c: Congruent, i: Incongruent), current trial congruency (C: Congruent, I: Incongruent), and group. The combination of lowercase and uppercase letters (e.g., cC) indicates current trial congruency (C: congruent, I: incongruent) preceded by a congruent (c) or an incongruent (i) previous trial.

RT	Cognitive Simon task								Emotional Simon task							
	Sad face block				Neutral face block				Sad face block				Neutral face block			
	cC	cI	iC	iI	cC	cI	iC	iI	cC	cI	iC	iI	cC	cI	iC	iI
Ideators	557.69 (117)	584.44 (102)	551.47 (101)	578.89 (98)	556.38 (100)	593.78 (107)	565.31 (109)	585.24 (99)	689.99 (97)	723.41 (104)	697.91 (106)	715.69 (90)	706.66 (113)	717.35 (95)	707.12 (99)	711.11 (95)
Attempters	540.75(116)	575.82 (121)	545.39 (110)	563.89 (114)	541.39 (112)	578.68 (118)	553.11 (116)	566.55 (115)	688.55 (114)	707.15 (98)	688.17 (109)	707.30 (103)	699.67 (106)	719.03 (103)	705.69 (107)	715.88 (98)
Controls	518.48 (77)	556.75 (81)	521.13 (70)	550.03 (76)	517.49 (81)	557.51 (85)	521.96 (77)	550.78 (84)	675.14 (95)	699.45 (97)	692.43 (95)	698.23 (96)	679.07 (92)	712.32 (96)	688.04 (96)	704.48 (99)
Percent error	Cognitive Simon task								Emotional Simon task							
	Sad face block				Neutral face block				Sad face block				Neutral face block			
	cC	cI	iC	iI	cC	cI	iC	iI	cC	cI	iC	iI	cC	cI	iC	iI
Ideators	4.46 (6)	6.37 (7)	5.76 (6)	4.76 (5)	3.86 (4)	5.73 (6)	4.95 (6)	6.19 (8)	10.13 (8)	10.36 (8)	8.49 (7)	8.42 (7)	11.03 (7)	12.95 (9)	12.02 (8)	13.75 (11)
Attempters	3.92 (4)	6.26 (6)	3.65 (4)	5.01 (5)	3.47 (5)	6.16 (6)	4.22 (5)	4.01 (5)	7.48 (8)	12.19 (11)	8.18 (8)	12.00 (11)	10.84 (8)	15.86 (9)	12.08 (7)	14.24 (10)
Controls	3.01 (5)	7.41 (7)	2.43 (4)	5.65 (5)	4.02 (5)	8.53 (6)	3.19 (4)	6.32 (6)	8.27 (7)	10.32 (9)	7.64 (8)	9.93 (8)	10.26 (7)	12.88 (8)	14.16 (9)	11.35 (8)

The main effect of task was significant, *F*(1, 134) = 696.74, *p* < .001, *MSe* = 16,426, $\eta_{p}^{2}$ = .84, with the cognitive task (*M* = 555 ms) performed faster than the emotional task (*M* = 702 ms). The main effect of block was also significant, *F*(1, 134) = 6.21, *p* = .01, *MSe* = 2,381, $\eta_{p}^{2}$ = .04, with sad blocks (*M* = 626 ms) performed faster than neutral blocks (*M* = 631 ms). Finally, the main effect of current trial congruency was significant, *F*(1, 134) = 127.00, *p* < .001, *MSe* = 2,324, $\eta_{p}^{2}$ = .49. A significantly longer mean RT on incongruent trials (*M* = 641 ms) than congruent trials (*M* = 617 ms) indicates a congruency effect [26].

The interaction between task and current trial congruency was significant, *F*(1, 134) = 16.59, *p* < .001, *MSe* = 1,075, $\eta_{p}^{2}$ = .11. To observe how the size of the congruency effect differs between the two tasks, separate analyses were conducted for each task with current trial congruency as a factor. Results showed that both the difference between incongruent (*M* = 570 ms) and congruent (*M* = 541 ms) trials for the cognitive task, *F*(1, 136) = 146.00, *p* < .001, *MSe* = 392, $\eta_{p}^{2}$ = .52, and the difference between incongruent (*M* = 711 ms) and congruent (*M* = 693 ms) trials for the emotional task, *F*(1, 136) = 47.09, *p* < .001, *MSe* = 452, $\eta_{p}^{2}$ = .26, were significant, but the difference was larger for the cognitive task resulting in a significant interaction. Unlike other congruency tasks, the size of the congruency effect in Simon tasks tends to increase with faster RTs [56]. The cognitive task was performed significantly faster than the emotional task. Accordingly, the congruency effect was larger for the cognitive task possibly due to its faster RT. Consistently, an additional correlation analysis between the RTs of cognitive and emotional tasks and the congruency effects of cognitive and emotional tasks showed a significant negative correlation, *r*(272) = -.16, *p* = .003, supporting that the cognitive task had a larger congruency effect in association with its faster RT.

The interaction between previous and current trial congruency was also significant, *F*(1, 134) = 24.90, *p* < .001, *MSe* = 772, $\eta_{p}^{2}$ = .16. It indicates a CSE when the interaction is significant due to the magnitude of the congruency effect being significantly smaller for current trials whose previous trial was incongruent rather than congruent [27]. Thus, the congruency effect was compared for each previous trial congruency. The size of the congruency effect was reduced after incongruent trials (17 ms), *F*(1, 136) = 54.10, *p* < .001, *MSe* = 377, $\eta_{p}^{2}$ = .29, compared to after congruent trials (29 ms), *F*(1, 136) = 149.95, *p* < .001, *MSe* = 392, $\eta_{p}^{2}$ = .52, indicating a CSE.

**Fig 2:**
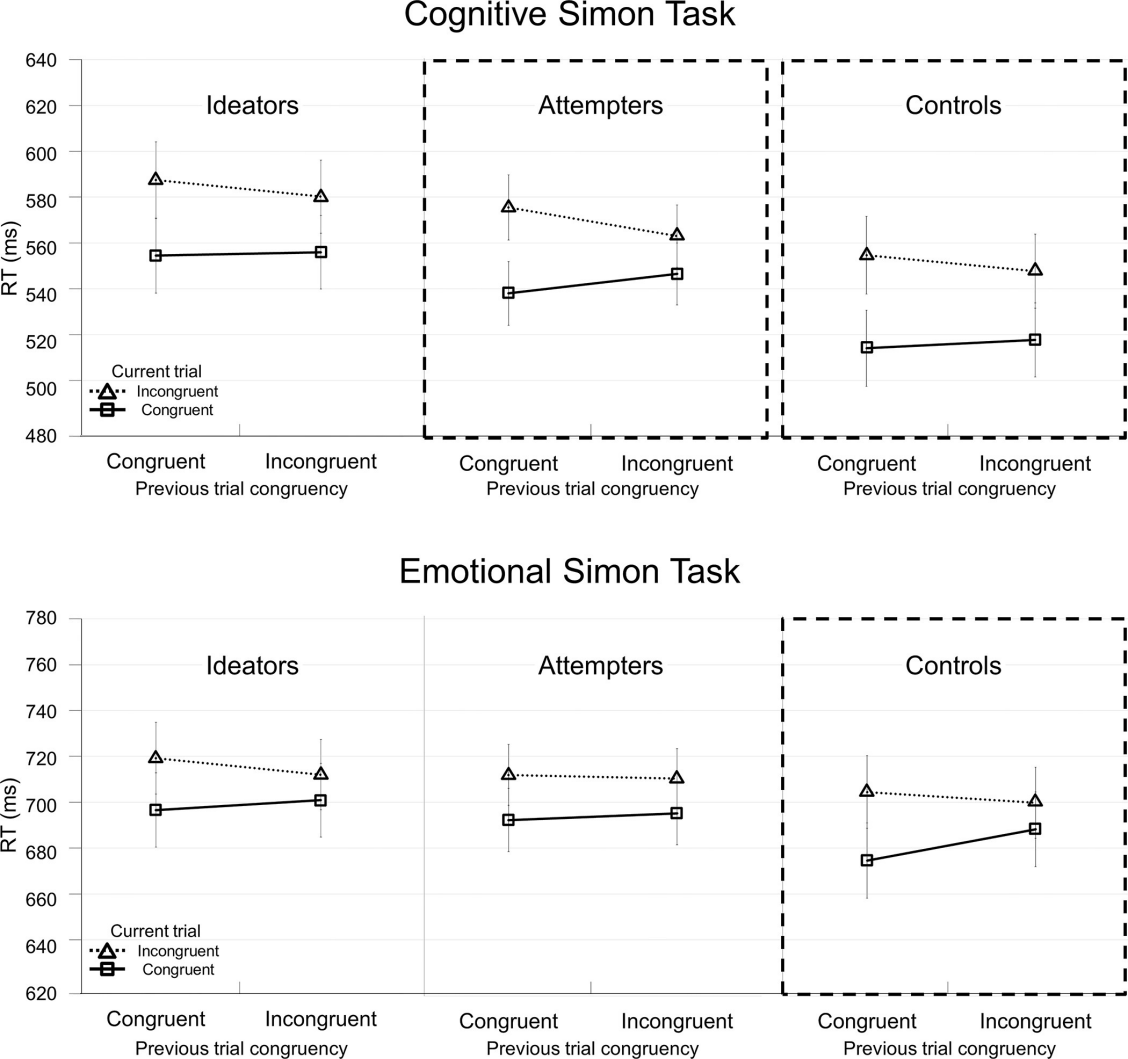
Mean RTs as a function of previous and current trial congruency for the cognitive and emotional Simon tasks across the three groups. When the interaction between previous trial congruency and current trial congruency is significant because the difference in mean RTs between incongruent and congruent trials (i.e., congruency effect) is significantly smaller following incongruent than congruent trials, the results demonstrate a CSE. The graphs outlined with dashed lines indicate the groups that showed a statistically significant CSE. Controls displayed significant CSE in both cognitive and emotional Simon tasks, whereas attempters showed only a significant CSE in the cognitive Simon task. Ideators showed a marginally significant CSE in both tasks. Pairwise comparisons between two groups showed that only between attempters and controls did the CSE between the two tasks significantly differ because the CSE was significantly smaller in the emotional compared to cognitive task for attempters, but the CSEs did not differ between the tasks for controls. Error bars show standard error of the mean across participants.

In evaluating our hypothesis that the CSEs in the cognitive task and the emotional task would differ between suicidal and nonsuicidal individuals, we examined the four-way interaction of group, task, previous and current trial congruency. Its significance implies that the CSE for each task differs across the groups. The interaction was marginally significant, *F*(2, 134) = 2.84, *p* = .06, *MSe* = 662, $\eta_{p}^{2}$ = .04. This was also the case when the sum score of PHQ-9 was incorporated as a covariate, *F*(2, 133) = 2.55, *p* = .08, *MSe* = 666, $\eta_{p}^{2}$ = .04. Further analyses on the mixed ANOVA results of the marginally significant four-way interaction were conducted separately for each group with task, previous trial congruency, and current trial congruency as three variables to examine differences across the groups. For controls, as expected, a significant CSE was observed, *F*(1, 39) = 13.06, *p* = .001, *MSe* = 299, $\eta_{p}^{2}$ = .25, and the three-way interaction was not significant, *F*(1, 39) < 1, indicating that comparable sizes of the CSE were obtained between the cognitive and emotional tasks. For ideators, the CSE was marginally significant, *F*(1, 39) = 3.37, *p* = .07, *MSe* = 567, $\eta_{p}^{2}$ = .08, and the three-way interaction was not significant, *F*(1, 39) < 1, indicating marginally significant CSEs in both tasks. Finally for attempters, the CSE was significant, *F*(1, 56) = 13.42, *p* = .001, *MSe* = 320, $\eta_{p}^{2}$ = .19, with the three-way interaction also being significant, *F*(1, 56) = 6.18, *p* = .02, *MSe* = 291, $\eta_{p}^{2}$ = .10. As predicted, the CSE was significant for the cognitive task, *F*(1, 56) = 22.38, *p* < .001, *MSe* = 260, $\eta_{p}^{2}$ = .29, whereas it was not for the emotional task, *F*(1, 56) < 1, indicating that attempters differed in reactive control between the cognitive task and the emotional task (Fig 2).

In contrast, the five-way interaction of group, task, block, previous and current trial congruency was not significant, *F*(2, 134) < 1, indicating that facial expressions did not modulate how the CSEs of the cognitive and emotional tasks differed across the groups. The four-way interaction of group, task, block, and current trial congruency was marginally significant, *F*(2, 134) = 2.93, *p* = .06, *MSe* = 719, $\eta_{p}^{2}$ = .04. No other main effects and interactions were significant or marginally significant (see Table 3).

**Table 3:** Statistics of the ANOVA results as a function of group, task, block, previous trial congruency (n1cong), and current trial congruency (n0cong).

	Statistics				Statistics		
	*F*	*p*	*η*_*p*_^*2*^		*F*	*p*	*η*_*p*_^*2*^
Group	.77	.47	.01	Group×Task×Block×n1cong	.23	.79	.003
Task	696.7	.000	.84	Task×n0cong	16.59	.000	.11
Block	6.21	.01	.04	Group×Task×n0cong	.25	.78	.004
n1cong	.10	.76	.00	Block×n0cong	.46	.50	.003
n0cong	127.0	.000	.49	Group×Block×n0cong	1.75	.18	.03
Group×Task	.97	.38	.01	Task×Block×n0cong	.94	.33	.007
Group×Block	.57	.57	.01	Group×Task×Block×n0cong	2.93	.06	.04
Group×n1cong	.41	.66	.01	n1cong×n0cong	24.9	.000	.16
Group×n0cong	.68	.51	.01	Group×n1cong×n0cong	.23	.79	.003
Task×Block	1.29	.26	.01	Task×n1cong×n0cong	.19	.67	.001
Group×Task×Block	1.52	.22	.02	Group×Task×n1cong×n0cong	2.84	.06	.04
Task×n1cong	1.57	.21	.01	Block×n1cong×n0cong	.93	.34	.007
Group×Task×n1cong	.23	.79	.003	Group×Block×n1cong×n0cong	.30	.74	.004
Block×n1cong	.01	.91	.00	Task×Block×n1cong×n0cong	.67	.42	.005
Group×Block×n1cong	.59	.56	.009	Group×Task×Block×n1cong×n0cong	.61	.54	.009
Task×Block×n1cong	1.65	.20	.01				

*Note*. *dfs*: (1, 134) for within-subject effects and (2, 134) for between-subject effects

We conducted pairwise comparisons between two groups to understand better how the CSEs differed between the two tasks across the groups. That is, ANOVAs were rerun with two groups instead of three. This additional analysis was to investigate how suicidal (attempters or ideators) and nonsuicidal controls may differ and how attempters and ideators may differ. In particular, comparing attempters and ideators is of import to investigate what risk factors distinguish people who enact suicide from people who only think of it, considering the ideation-to-action framework of identifying moderating factors in the progression of ideation to action [3–5]. Results indicated that the interaction of task, previous trial congruency, current trial congruency, and group was significant in the comparison between attempters and controls, *F*(1, 95) = 5.02, *p* = .03, *MSe* = 633, $\eta_{p}^{2}$ = .05, marginally significant in the comparison between attempters and ideators, *F*(1, 95) = 3.18, *p* = .08, *MSe* = 643, $\eta_{p}^{2}$ = .03, but not in the comparison between ideators and controls, *F*(1, 78) < 1 (see Table 4).

**Table 4:** Statistics of the pairwise comparison of the CSE.

	Attempters vs. Controls			Ideators vs. Controls			Attempters vs. Ideators		
	*F*	*p*	*η*_*p*_^*2*^	*F*	*p*	*η*_*p*_^*2*^	*F*	*p*	*η*_*p*_^*2*^
Group×CSE	.11	.74	.001	.41	.53	.005	.17	.68	.002
Group×task×CSE	5.02	.03	.50	.15	.70	.002	3.18	.08	.03
Group×CSE in Emotional task	2.69	.10	.03	.50	.48	.006	.74	.39	.008
Group×CSE in Cognitive task	2.42	.12	.03	.05	.83	.001	2.22	.14	.02

*Note*. CSE indicates the interaction between previous and current trial congruency. [*df*s: (1, 95) for comparison between attempters and controls; (1, 78) for comparison between ideators and controls; (1, 95) for comparison between attempters and ideators]

We also conducted pairwise comparisons between two groups with the sum score of PHQ-9 as a covariant. Results showed that the three-way interaction was significant between attempters and controls, *F*(1, 94) = 4.29, *p* = .04, *MSe* = 638, $\eta_{p}^{2}$ = .05, marginally significant between attempters and ideators, *F*(1, 94) = 3.61, *p* = .06, *MSe* = 647, $\eta_{p}^{2}$ = .04, and not significant between ideators and controls, *F*(1, 77) < 1.

#### Percent error data

We also conducted a mixed ANOVA on the percent error data with task, block, previous trial congruency, and current trial congruency as within-subject variables and group as a between-subject variable. The RT and percent error results were not consistent as we did not observe a significant or a marginally significant four-way interaction of group, task, previous trial congruency, and current trial congruency in the percent error data. However, it is often the case that CSE analyses focus on mean RTs whereas error rates are only considered for ruling out speed-accuracy trade-offs due to accuracy results displaying ceiling effects [e.g., 57]. We did not observe a significant positive correlation between RT and accuracy in any of the experimental conditions (-.31≤ Spearman’s *r(135)* ≤ .07), and the overall mean accuracy was .92.

Although we did not observe a significant group difference between the two tasks in CSE, we observed a significant group difference between the two tasks in congruency effect in the percent error data, which consistently support our main hypothesis that suicidal group shows poorer control in the emotional task but not in the cognitive task.

The main effect of task was significant, *F*(1, 134) = 155.24, *p* < .0001, *MSe* = 126.11, $\eta_{p}^{2}$ = .54, as the cognitive task was performed with lower percent error (5%) than the emotional task (11%). The main effect of block was also significant, *F*(1, 134) = 57.85, *p* < .001, *MSe* = 25.61, $\eta_{p}^{2}$ = .30, as sad face blocks were performed with lower percent error (7%) than the neutral face blocks (9%). Finally, the main effect of current trial congruency was significant, *F*(1, 134) = 45.28, *p* < .001, *MSe* = 49.42, $\eta_{p}^{2}$ = .25, as congruent trials were performed with lower percent error (7%) than incongruent trials (9%), indicating a congruency effect. No other main effects were significant.

**Fig 3:**
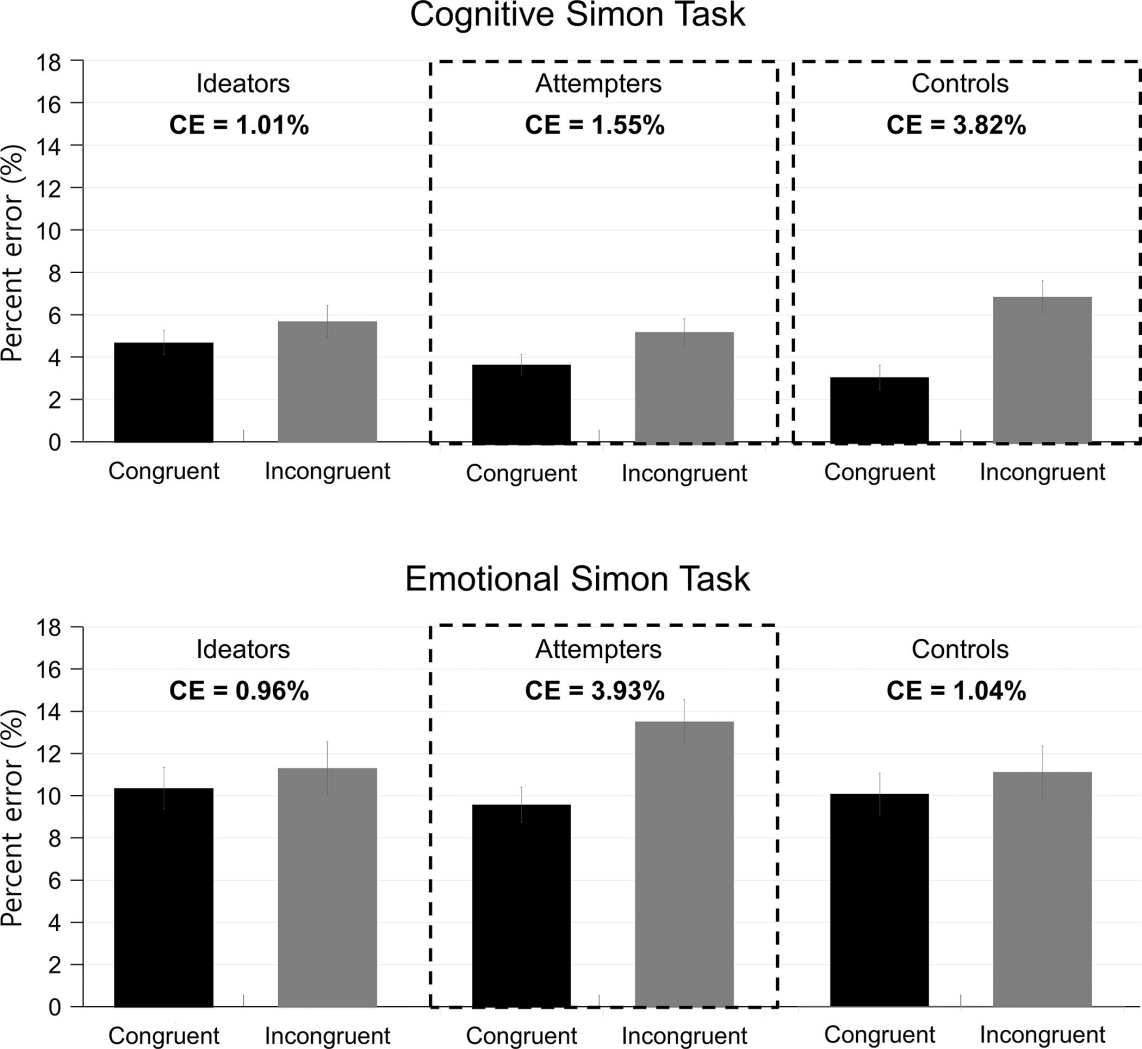
Mean percent error as a function of current trial congruency for the cognitive and emotional Simon tasks across the three groups. The congruency effect (CE) is calculated as the difference in percent error between incongruent trials and congruent trials. The larger the magnitude of the CE is, the greater the interference by task-irrelevant information is. The graphs outlined with dashed lines indicate the groups that showed a statistically significant CE. Whereas the CE was not significant in both tasks for ideators, it was for attempters. The CE was also significantly larger in the emotional Simon task than in the cognitive Simon task for attempters. The CE was only significant in the cognitive Simon task for nonsuicidal controls. Error bars show standard error of the mean across participants.

The three-way interaction of group, task, and current trial congruency was significant, indicating that the groups differed in how much interference participants experienced (i.e., congruency effect) in the two tasks, *F*(1, 134) = 9.47, *p* < .001, *MSe* = 33.20, $\eta_{p}^{2}$ = .12. Further analyses conducted for each group with task and current trial congruency as variables showed that the interaction was significant for the attempters, *F*(1, 56) = 8.76, *p* = .005, *MSe* = 9.23, $\eta_{p}^{2}$ = .14, and for the controls, *F*(1, 39) = 12.41, *p* = .001, *MSe* = 6.24, $\eta_{p}^{2}$ = .24, but not for the ideators, *F* (1, 39) < 1, indicating that the congruency effect did not differ between the two tasks for ideators. Separate analysis conducted for attempters with current trial congruency as a variable showed that the congruency effect was significant in the cognitive Simon task, *F*(1, 56) = 12.29, *p* < .001, *MSe* = 5.56, $\eta_{p}^{2}$ = .18. The congruency effect was also significant in the emotional Simon task but the effect was significantly larger, *F*(1, 56) = 20.68, *p* < .001, *MSe* = 14.83, $\eta_{p}^{2}$ = .35. For controls, the congruency effect was significant in the cognitive task, *F*(1, 39) = 32.02, *p* < .001, *MSe* = 9.10, $\eta_{p}^{2}$ = .45, but not in the emotional task, *F*(1, 39) = 2.16, *p* = .15. In sum, ideators were less affected by distractor interference in both cognitive and emotional tasks, whereas attempters showed distractor interference in both tasks. Interestingly, attempters were more affected by distractor interference in emotional task than in cognitive task. By contrast, controls showed distractor interference only in the cognitive task but not in the emotional task (Fig 3).

### Group differences in self-reported cognitive flexibility

Significant group differences were observed in the CFI total, *F*(2, 134) = 15.26, *p* < .001, *MSe* = 203, $\eta_{p}^{2}$ = .186, the Alternatives subscale, *F*(2, 134) = 5.82, *p* = .004, *MSe* = 82, $\eta_{p}^{2}$ = .080, and the Control subscale, *F*(2, 134) = 19.27, *p* < .001, *MSe* = 60, $\eta_{p}^{2}$ = .223. With higher scores indicating greater cognitive flexibility, both attempters and ideators had significantly lower scores than controls. Post-hoc Tukey HSD tests showed that the difference in the CFI total between controls and attempters, *p* < .001, Cohen’s *d* = .95, and between controls and ideators were significant, *p* < .001, Cohen’s *d* = 1.28, but not between attempters and ideators, *p* = .88, Cohen’s *d* = .10. Post-hoc Tukey HSD results on the CFI subscales consistently showed that the difference was significant between controls and attempters (Alternatives: *p* = .02, Cohen’s *d* = .56; Control: *p* < .001, Cohen’s *d* = 1.11) and between controls and ideators (Alternatives: *p* < .001, Cohen’s *d* = .82; Control: *p* < .001, Cohen’s *d* = 1.35) but not between attempters and ideators (Alternatives: *p* = .81, Cohen’s *d* = .13; Control: *p* = .99, Cohen’s *d* = .03).

#### Correlation of CSE and self-reported cognitive flexibility

To examine linkages between reactive control and cognitive flexibility, we performed correlation analyses between each participant’s RT CSE and their CFI scores (Total, Alternatives, and Control). The analyses were first performed including all participants regardless of their assigned groups and then for each group. We ran Spearman’s correlation coefficient, right sided, expecting a positive correlation. We first calculated correlation coefficients between the total CSE scores (across all tasks and blocks) and CFI. Only the attempters showed a significant correlation between the CSE and the CFI Control subscale, *r*(55) = .31, *p* = .001. However, this did not survive false discovery rate (FDR) multiple comparison correction. We next calculated Spearman’s correlation between each task’s CSE and CFI. Again, only attempters showed a significant correlation between cognitive Simon task CSEs and Control subscales, *r*(55) = .31, *p* = .01, which also did not survive FDR correction. Finally, the correlation coefficients between CSEs, calculated as a function of task and block, and CFI scores were calculated. No significant correlations were found.

## Discussion

We investigated the cognitive ability to control interference in suicidal and nonsuicidal individuals. We focused on their ability to inhibit distractions in conflicting situations because we hypothesized that failing to manage distractions upon facing conflict may explain the cognitive processing of why suicidal individuals would feel urges to attempt suicide in stressful and conflicting situations. We expected that the control of interference would be poorer in suicidal individuals when the interference is instigated by task-irrelevant negative emotional stimuli. Our main findings from behavioral and self-reported measures are as follows: (a) Attempters showed differences from controls in adjusting reactive control during the emotional Simon task but not during the cognitive Simon task. (b) Attempters were more interrupted by task-irrelevant information in emotional Simon task than in the cognitive Simon task, contrastingly to controls and ideators. (c) Ideators were not interrupted by task-irrelevant information in both Simon tasks. (d) Ideators and attempters showed impaired cognitive flexibility compared to controls in the self-report measure. (e) The group difference in the Simon tasks persisted even when covarying for depressive severity symptoms.

Our main hypothesis was that suicidal individuals would experience susceptibility to interference and difficulty in reinforcing reactive control to deal with conflict. Accordingly, it was predicted that suicidal and nonsuicidal individuals would show a significant group difference in CSE as suicidal individuals would show a significantly smaller CSE in a Simon task. To examine in which circumstances this group difference would be displayed, we hypothesized that the conflict generated by human faces rather than simple squares would impair reactive control. We predicted that if suicidal individuals would be more impaired in resolving conflict generated by negative emotional faces than by neutral faces, their CSE would be smaller in sad compared to neutral blocks. Our hypotheses were partially supported because while attempters showed non-significant CSE only in the emotional task, their performance did not differ between sad and neutral blocks.

Even though we predicted that negative emotion would affect the impairment of reactive control for suicidal individuals, the variable, block, did not modulate the difference in CSEs between the two tasks. Nonetheless, it is possible that the neutral faces were perceived negatively in a biased manner due to the sad faces. Whereas the discrete-category view of emotion suggests that a face displays an expression that belongs to a single emotional category [58], the dimensional view suggests that facial expressions are dimensional and cannot be divided into discrete categories [59]. Therefore, individuals can perceive a facial expression more or less positively or negatively depending on the face’s embedded emotional contexts [60]. Even though only half of the faces were sad during the emotional task, participants possibly perceived the neutral faces more negatively because of the sad faces. Instead, the red and yellow squares may have been perceived more explicitly as neutral stimuli compared to the faces.

We do not suggest directionality that emotion caused impairment of reactive control in attempters. Future studies are necessary to directly manipulate mood or emotional contexts to suggest causal relationships of how emotion changes reactive control. In manipulating emotional contexts, their relevance to the task may play a critical role. In our study, as participants responded to the biological sex of the faces rather than to the facial expressions, emotional information was task-irrelevant. Examining reactive control when emotional information is task-irrelevant was based on our assumption that suicidal individuals would be vulnerable to distractions upon facing conflict instigated by negative emotional stimuli. However, a context when emotional information is task-relevant should also be considered as previous research demonstrated that relevance determines the extent of influence emotion has on information processing; the extent is limited to early perceptual stages when emotional information is task-irrelevant [61]. Therefore, future studies manipulating emotional contexts should consider the relevance of emotional information to task goals.

The congruency effect results from distractor interference [14, 26], and the CSE is the significant reduction of the congruency effect after experiencing conflict [27]. Our results showed that emotion was critical in both the congruency effect and the CSE to distinguish attempters from the two groups. Attempters showed a significantly larger congruency effect in the emotional Simon task compared to the cognitive Simon task, indicating that attempters experienced more interference in the emotional compared to the cognitive task. They also did not display a CSE only in the emotional task, resulting in a significant group effect that distinguished attempters from controls. This indicates that attempters experienced more difficulty to adjust reactive control in the emotional task contrary to controls, supporting our hypothesis that reactive control in negative emotional context can distinguish attempters from nonsuicidal controls. This group difference was retained even after controlling for current depressive symptoms.

Attempters not displaying a CSE during the processing of human faces, contrary to controls, imply that they were more disturbed by conflict with less efficiency in resolving conflict. This interpretation is based on our examination of how task-irrelevant representations of a face disturbed the goal of successfully performing the task. On the other hand, a scenario in which suicidal behavior is the goal can be considered. In this case, inefficient reactive control in contexts with strong emotion can be an adaptive strategy for suicidal individuals, because reinforcing reactive control to facilitate goal achievement could encourage suicidal behaviors. If inflexibility to respond to interfering information in negative emotional states is an adaptive strategy for suicidal individuals, how it is developed and how it progresses with the severity of suicidal behaviors would be interesting research topics to investigate in future studies.

While attempters and controls significantly differed in CSE during the emotional Simon task, one may question if the non-significant CSE in attempters can truly be considered a deficit compared to controls. Studies exist which interpret larger CSEs as flexibility and improved deployment of control [24, 62], but a larger CSE can be alternatively considered a neglect or inefficiency of proactive control [63]. Whereas reactive control is transient reactivation of a goal to resolve interference upon detecting conflict, proactive control is sustained maintenance of goal representations [22]. If the CSE is large, it could indicate that participants were not actively engaged in maintaining task sets and thus were unprepared for conflict. In this case, a significantly larger CSE for controls compared to attempters in our findings can be interpreted as controls implementing attentional control when triggered upon facing conflict as they were less proactive about task sets. For attempters, on the other hand, a non-significant CSE could be interpreted as relying more on proactive instead of reactive control. However, we view attempters’ non-significant CSE as an inefficiency in reactive control considering their congruency effect results. Attempters showed a significantly larger congruency effect during the emotional compared to the cognitive task. This suggests that attempters were more interrupted by distractions during the emotional task. This interruption was not efficiently resolved as the CSE was not significant in the emotional task. In contrast, the congruency effect was significant in the cognitive task but not in the emotional task for controls, yielding a significant difference between the two tasks. This indicates that they were less interrupted by distractions during the emotional task with successful implementation of the task goal. Thus, the relatively larger CSE of controls in the emotional task is less likely to be interpreted that they were not actively engaged in maintaining task sets and inhibiting distractions. Still, to investigate whether the significant group difference is due to poorer reactive control in attempters or poorer proactive control in controls, a task design that manipulates the proportion of the congruent items in future studies would be necessary [64].

We categorized suicidal individuals into ideators and attempters to examine if the severity of adjusting reactive control differentiates the two groups. Interestingly, only the ideators showed non-significant congruency effects in both tasks. This indicates that ideators were less interfered by distractions. While ideators did not differ significantly from attempters or controls in CSE, ideators could be considered more similar to controls than attempters because of the non-significant difference between ideators and controls in the pairwise comparisons. In addition, the ANOVA results of ideators indicated marginally significant CSEs in both tasks. Accordingly, our findings can be interpreted as ideators being good at controlling interference and only weakly impaired in adjusting reactive control in contrast to attempters. This possibility may explain why ideators think of suicide but do not enact suicide. Nonetheless, the self-report results indicated that ideators and attempters both scored more poorly on cognitive flexibility than controls. Accordingly, if ideators are considered more similar to attempters than controls, the task results imply more inadequate reactive control for ideators than attempters as they showed a marginally significant CSE even in the cognitive task. Our data are insufficient to test these possibilities. Rather, considering the pairwise comparison results that ideators did not significantly differ from either group, ideators possibly share characteristics with attempters and controls, indicating their stronger heterogeneity than the other groups. You et al. [39] also reported that ideators and attempters did not differ significantly in implementing inhibitory regulation during a stop-signal task. While the severity of suicidal behaviors may exist on a continuum from ideation to action [3–5], participants are assigned into discrete groups in research studies that examine group effects. During this assignment, a participant, for example, who has never made an attempt but is in a preliminary stage, would be categorized as an ideator, while the person may share more characteristics with attempters than ideators. Accordingly, although distinguishing ideators from attempters to compare their regulatory functions is a necessary step to examine factors that moderate the progression from ideation to attempt, empirical evidence is yet unclear.

As examining the CSE has demonstrated significant group differences between attempters and controls, one may question if it provides a reliable index to be used as a clinical measure to determine potential suicidal risk. The CSE is a reliable experimental effect that is highly replicated with most participants within an experiment showing the effect [65]. This index of conflict adaptation is also stable as demonstrated by reliable internal consistency and 2-week test-retest stability [66, 67]. However, correlational research that examined if individual differences in laboratory measures of cognitive control predict self-control success outside the lab has shown inconsistency [68, 69]. Especially, recent studies demonstrated that the difference score metrics used in congruency tasks contain large error variance, which leads to poor reliability in correlational research [70, 71]. Accordingly, we expect the use of CSE to predict individual differences in suicidal risks to be reliable only when precise individual-level estimates of the effect can be obtained, such as by having a larger number of trials to reduce within-subject variance [72, 73]. There have also been many approaches to improve the reliability of difference score measures of cognitive control, such as Bayesian hierarchical modeling [74] and generative modeling [75]. We expect further examination is necessary in deciding on using the CSE for clinical applications.

A couple of differences existed between the self-report and behavioral results. The correlation analyses between the CFI scores and the CSE did not show significant positive correlations. Inconsistency also existed particularly for ideators between their self-report and task performance. Whereas they reported poorer cognitive flexibility than controls, their task performance did not differ significantly from controls. You et al. [39] also reported that self-report and task-based measures of regulatory functions were not significantly correlated. Brokke et al. [15] also reported that whereas attention control was worse for attempters than ideators, self-report results did not differ between the two groups. These findings suggest that the perception of themselves does not necessarily correspond to their actual performance. Another explanation for the inconsistency between task and self-report measures is that experimental effects, including the CSE, require more precise individual estimates for correlational research than for experimental research [71, 72, 74]. The CSE is reliable in experimental research, which manipulates within-subject effects and compares across conditions of group averaged scores, but for conducting correlational research to characterize individual differences and to find relations across multiple measures, more precise individual estimates are required [71].

Limits on the generalizability of our findings exist owing to a narrow age range from 19 to 32. As much as young adults, the elderly above 70 have high suicide rates [76], and aging is associated with impairment in regulatory functions [77]. Hence, the elderly may display differences from young adults. Our sample is also limited in that a high proportion of the participants were university attending when the level of education is a known factor related to suicide [78, 79] and cognitive control [80–82]. Considering that higher educational attainment positively affects cognitive control [81], suicidal individuals with lower levels of education may display differences in reactive control compared to our sample. Our study also failed to include information about the recency of suicidal behavior. We assigned participants into groups based on their lifetime histories of suicidal ideation or attempt but did not include the information on their recency in the assessment. To capture what is present during or proximal to suicidal episodes, limiting to more recently conducted behaviors would be necessary for future research. Finally, our study is limited for not comparing with a nonsuicidal clinical group, such as individuals with major depressive disorder. Depression is known to be one of the highest risk factors for suicide ideation [83]. The current findings should be cautiously interpreted considering these limitations.

## Conclusion

By comparing performance between the cognitive and emotional tasks, we demonstrated a clear difference in attempters from controls: attempters showed more interference by distractions and poorer adjustment of reactive control in the emotional than in the cognitive task. This impairment, restricted to the task that used human faces as task stimuli, implies suicide attempts are likely associated with difficulty in managing conflict in dealing with distractions related to people and psychosocial stress. Furthermore, our self-report results consistently indicated impaired cognitive flexibility in attempters compared to controls. In conclusion, our findings suggest that the inefficiency in controlling interference and resolving conflict may be a key cognitive factor that distinguishes suicide attempters from nonsuicidal controls.

## References

[1] Franklin JC, Ribeiro JD, Fox KR, Bentley KH, Kleiman EM, Huang X, et al. Risk factors for suicidal thoughts and behaviors: A meta-analysis of 50 years of research. Psychol Bull [Internet]. 2017;143(2):187–232. Available from: doi: 10.1037/bul0000084 27841450

[2] Turecki G, Brent DA, Gunnell D, O’Connor RC, Oquendo MA, Pirkis J, et al. (2019). Suicide and suicide risk. Nature reviews Disease primers [Internet]. 2019; 5(1): 74. Available from: https://www.nature.com/articles/s41572-019-0121-0 doi: 10.1038/s41572-019-0121-0 31649257

[3] Joiner TE. Why people die by suicide. London, England: Harvard University Press; 2009.

[4] Klonsky ED, May AM. Differentiating suicide attempters from suicide ideators: a critical frontier for suicidology research. Suicide Life Threat Behav [Internet]. 2014;44(1):1–5. Available from: doi: 10.1111/sltb.12068 24313594

[5] O’Connor RC. The integrated motivational-volitional model of suicidal behavior. Crisis [Internet]. 2011;32(6):295–8. Available from: doi: 10.1027/0227-5910/a000120 21945841

[6] Liu RT, Miller I. Life events and suicidal ideation and behavior: a systematic review. Clin Psychol Rev [Internet]. 2014;34(3):181–92. Available from: doi: 10.1016/j.cpr.2014.01.006 24534642

[7] O’Connor RC, Nock MK. The psychology of suicidal behaviour. Lancet Psychiatry [Internet]. 2014;1(1):73–85. Available from: doi: 10.1016/S2215-0366(14)70222-6 26360404

[8] Wenzel A, Beck AT. A cognitive model of suicidal behavior: Theory and treatment. Appl Prev Psychol [Internet]. 2008;12(4):189–201. Available from: 10.1016/j.appsy.2008.05.001

[9] Hausman C, Meffert BN, Mosich MK, Heinz AJ. Impulsivity and cognitive flexibility as neuropsychological markers for suicidality: A multi-modal investigation among military veterans with alcohol use disorder and PTSD. Arch Suicide Res [Internet]. 2020;24(3):313–26. Available from: doi: 10.1080/13811118.2019.1635930 31248349 PMC6954988

[10] Miranda R, Gallagher M, Bauchner B, Vaysman R, Marroquín B. Cognitive inflexibility as a prospective predictor of suicidal ideation among young adults with a suicide attempt history: Cognitive Inflexibility and Suicidal Behavior. Depress Anxiety [Internet]. 2012;29(3):180–6. Available from: 10.1002/da.20915 22147587

[11] Pollock LR, Williams JMG. Problem-solving in suicide attempters. Psychol Med [Internet]. 2004;34(1):163–7. Available from: doi: 10.1017/s0033291703008092 14971637

[12] Kane MJ, Engle RW. Working-memory capacity and the control of attention: the contributions of goal neglect, response competition, and task set to Stroop interference. J Exp Psychol Gen [Internet]. 2003;132(1):47–70. Available from: doi: 10.1037/0096-3445.132.1.47 12656297

[13] Maruff P, Danckert J, Camplin G, Currie J. Behavioral goals constrain the selection of visual information. Psychol Sci [Internet]. 1999;10(6):522–5. Available from: 10.1111/1467-9280.00199

[14] Stroop JR. Studies of interference in serial verbal reactions. J Exp Psychol [Internet]. 1935;18(6):643–62. Available from: 10.1037/h0054651

[15] Brokke SS, Landrø NI, Haaland VØ. Cognitive control in suicide ideators and suicide attempters. Front Psychol [Internet]. 2020;11:595673. Available from: doi: 10.3389/fpsyg.2020.595673 33424712 PMC7785752

[16] Burton CZ, Vella L, Weller JA, Twamley EW. Differential effects of executive functioning on suicide attempts. J Neuropsychiatry Clin Neurosci [Internet]. 2011 Spring;23(2):173–9. Available from: doi: 10.1176/jnp.23.2.jnp173 21677246 PMC3626287

[17] Cha CB, Najmi S, Park JM, Finn CT, Nock MK. Attentional bias toward suicide-related stimuli predicts suicidal behavior. J Abnorm Psychol [Internet]. 2010;119(3):616–22. Available from: doi: 10.1037/a0019710 20677851 PMC2994414

[18] Richard-Devantoy S, Berlim MT, Jollant F. A meta-analysis of neuropsychological markers of vulnerability to suicidal behavior in mood disorders. Psychol Med [Internet]. 2014;44(8):1663–73. Available from: doi: 10.1017/S0033291713002304 24016405

[19] Stewart JG, Glenn CR, Esposito EC, Cha CB, Nock MK, Auerbach RP. Cognitive control deficits differentiate adolescent suicide ideators from attempters. J Clin Psychiatry [Internet]. 2017;78(6):e614–21. Available from: doi: 10.4088/JCP.16m10647 28199073

[20] Botvinick MM, Braver TS, Barch DM, Carter CS, Cohen JD. Conflict monitoring and cognitive control. Psychol Rev [Internet]. 2001;108(3):624–52. Available from: doi: 10.1037/0033-295x.108.3.624 11488380

[21] Botvinick MM, Cohen JD, Carter CS. Conflict monitoring and anterior cingulate cortex: an update. Trends Cogn Sci [Internet]. 2004;8(12):539–46. Available from: doi: 10.1016/j.tics.2004.10.003 15556023

[22] Braver TS. The variable nature of cognitive control: a dual mechanisms framework. Trends Cogn Sci [Internet]. 2012;16(2):106–13. Available from: doi: 10.1016/j.tics.2011.12.010 22245618 PMC3289517

[23] Stürmer B, Leuthold H, Soetens E, Schröter H, Sommer W. Control over location-based response activation in the Simon task: behavioral and electrophysiological evidence. J Exp Psychol Hum Percept Perform [Internet]. 2002;28(6):1345–63. Available from: doi: 10.1037//0096-1523.28.6.1345 12542132

[24] Yang Q, Pourtois G. Reduced flexibility of cognitive control: reactive, but not proactive control, underpins the congruency sequence effect. Psychol Res [Internet]. 2022;86(2):474–84. Available from: doi: 10.1007/s00426-021-01505-6 33779833

[25] Simon JR, Rudell AP. Auditory S-R compatibility: the effect of an irrelevant cue on information processing. J Appl Psychol [Internet]. 1967;51(3):300–4. Available from: doi: 10.1037/h0020586 6045637

[26] Simon JR. The effects of an irrelevant directional CUE on human information processing. In: Advances in Psychology. Elsevier; 1990. p. 31–86.

[27] Gratton G, Coles MG, Donchin E. Optimizing the use of information: strategic control of activation of responses. J Exp Psychol Gen [Internet]. 1992;121(4):480–506. Available from: doi: 10.1037//0096-3445.121.4.480 1431740

[28] Kim S, Lee SH, Cho YS. Control processes through the suppression of the automatic response activation triggered by task-irrelevant information in the Simon-type tasks. Acta Psychol (Amst) [Internet]. 2015;162:51–61. Available from: doi: 10.1016/j.actpsy.2015.10.001 26479902

[29] Rustamov N, Rodriguez-Raecke R, Timm L, Agrawal D, Dressler D, Schrader C, et al. Absence of congruency sequence effects reveals neurocognitive inflexibility in Parkinson’s disease. Neuropsychologia [Internet]. 2013;51(14):2976–87. Available from: doi: 10.1016/j.neuropsychologia.2013.10.025 24212103

[30] Dennis JP, Vander Wal JS. The cognitive flexibility inventory: Instrument development and estimates of reliability and validity. Cognit Ther Res [Internet]. 2010;34(3):241–53. Available from: 10.1007/s10608-009-9276-4

[31] Ionescu T. Exploring the nature of cognitive flexibility. New Ideas Psychol [Internet]. 2012;30(2):190–200. Available from: 10.1016/j.newideapsych.2011.11.001

[32] Miranda R, Gallagher M, Bauchner B, Vaysman R, Marroquín B. Cognitive inflexibility as a prospective predictor of suicidal ideation among young adults with a suicide attempt history. Depression and anxiety [Internet]. 2012;29(3): 180–186. Available from: doi: 10.1002/da.20915 22147587

[33] Miranda R, Valderrama J, Tsypes A, Gadol E, Gallagher M. Cognitive inflexibility and suicidal ideation: Mediating role of brooding and hopelessness. Psychiatry research [Internet]. 2013;210(1):174–181. Available from: doi: 10.1016/j.psychres.2013.02.033 23528518 PMC6003697

[34] Rogers ML, Joiner TE. Suicide-specific rumination relates to lifetime suicide attempts above and beyond a variety of other suicide risk factors. Journal of Psychiatric Research [Internet]. 2018;98:78–86. Available from doi: 10.1016/j.jpsychires.2017.12.017 29304348

[35] Baca-Garcia E, Parra CP, Perez-Rodriguez MM, Sastre CD, Torres RR, Saiz-Ruiz J, et al. Psychosocial stressors may be strongly associated with suicide attempts. Stress Health [Internet]. 2007;23(3):191–8. Available from: 10.1002/smi.1137

[36] Osvath P, Vörös V, Fekete S. Life events and psychopathology in a group of suicide attempters. Psychopathology [Internet]. 2004;37(1):36–40. Available from: doi: 10.1159/000077018 14988649

[37] Stewart JG, Shields GS, Esposito EC, Cosby EA, Allen NB, Slavich GM, et al. Life stress and suicide in adolescents. J Abnorm Child Psychol [Internet]. 2019;47(10):1707–22. Available from: doi: 10.1007/s10802-019-00534-5 31028559 PMC6717522

[38] Dour HJ, Cha CB, Nock MK. Evidence for an emotion-cognition interaction in the statistical prediction of suicide attempts. Behav Res Ther [Internet]. 2011;49(4):294–8. Available from: doi: 10.1016/j.brat.2011.01.010 21353203

[39] You S, Lim CE, Park M, Ryu S, Lee HJ, Choi JM, et al. Response inhibition in emotional contexts in suicide ideators and attempters: Evidence from an emotional stop-signal task and self-report measures. Psychol Violence [Internet]. 2020;10(6):594–603. Available from: 10.1037/vio0000351

[40] Spitzer RL, Kroenke K, Williams JB. Validation and utility of a self-report version of PRIME-MD: the PHQ primary care study. Primary Care Evaluation of Mental Disorders. Patient Health Questionnaire. JAMA [Internet]. 1999;282(18):1737–44. Available from: doi: 10.1001/jama.282.18.1737 10568646

[41] Anderson SF, Kelley K, Maxwell SE. Sample-size planning for more accurate statistical power: A method adjusting sample effect sizes for publication bias and uncertainty. Psychol Sci [Internet]. 2017;28(11):1547–62. Available from: doi: 10.1177/0956797617723724 28902575

[42] Posner K, Brent D, Lucas C, Gould M, Stanley B, Brown G, et al. New York, NY: Columbia University; 2008.

[43] Posner K, Brown GK, Stanley B, Brent DA, Yershova KV, Oquendo MA, et al. The Columbia-Suicide Severity Rating Scale: initial validity and internal consistency findings from three multisite studies with adolescents and adults. Am J Psychiatry [Internet]. 2011;168(12):1266–77. Available from: doi: 10.1176/appi.ajp.2011.10111704 22193671 PMC3893686

[44] Jang H, Park E, Jon D, Park HJ, Hong HJ, Jung MH, et al. Validation of the Columbia suicide severity rating scale in depression patients. Korean J Clin Psychol [Internet]. 2014;33(4):799–814. Available from: 10.15842/kjcp.2014.33.4.008

[45] Burke TA, Hamilton JL, Ammerman BA, Stange JP, Alloy LB. Suicide risk characteristics among aborted, interrupted, and actual suicide attempters. Psychiatry Res [Internet]. 2016;242:357–64. Available from: doi: 10.1016/j.psychres.2016.05.051 27344029 PMC5247268

[46] Rogers ML, Hom MA, Dougherty SP, Gallyer AJ, Joiner TE. Comparing suicide risk factors among individuals with a history of aborted, interrupted, and actual suicide attempts. Arch Suicide Res [Internet]. 2020;24(sup1):57–74. Available from: doi: 10.1080/13811118.2018.1522283 30303461

[47] Centers for Disease Control and Prevention. Self-directed violence surveillance: Uniform definitions and recommended data elements: Version 1.0. Createspace; 2014.

[48] Husky MM, Olfson M, He J-P, Nock MK, Swanson SA, Merikangas KR. Twelve-month suicidal symptoms and use of services among adolescents: results from the National Comorbidity Survey. Psychiatr Serv [Internet]. 2012;63(10):989–96. Available from: doi: 10.1176/appi.ps.201200058 22910768 PMC5100004

[49] Kessler RC, Berglund P, Borges G, Nock M, Wang PS. Trends in suicide ideation, plans, gestures, and attempts in the United States, 1990–1992 to 2001–2003. JAMA [Internet]. 2005;293(20):2487–95. Available from: doi: 10.1001/jama.293.20.2487 15914749

[50] Nock MK, Green JG, Hwang I, McLaughlin KA, Sampson NA, Zaslavsky AM, et al. Prevalence, correlates, and treatment of lifetime suicidal behavior among adolescents: results from the National Comorbidity Survey Replication Adolescent Supplement. JAMA Psychiatry [Internet]. 2013;70(3):300–10. Available from: doi: 10.1001/2013.jamapsychiatry.55 23303463 PMC3886236

[51] Kim S-M, Kwon Y-J, Jung S-Y, Kim M-J, Cho YS, Kim HT, et al. Development of the Korean facial emotion stimuli: Korea University Facial Expression Collection 2nd edition. Front Psychol [Internet]. 2017;8:769. Available from: doi: 10.3389/fpsyg.2017.00769 28553255 PMC5427125

[52] Kim S, Cho YS. Congruency sequence effect without feature integration and contingency learning. Acta Psychol (Amst) [Internet]. 2014;149:60–8. Available from: doi: 10.1016/j.actpsy.2014.03.004 24704781

[53] Lim CE, Cho YS. Response mode modulates the congruency sequence effect in spatial conflict tasks: Evidence from aimed-movement responses. Psychological Research [Internet]. 2021;85: 2047–2068. Available from: https://hplab-ku.github.io/pdfs/papers/2021_lim_cho_1.pdf doi: 10.1007/s00426-020-01376-3 32592067

[54] Lee J, Cho YS. Congruency sequence effect in cross-task context: Evidence for dimension-specific modulation. Acta Psychologica [Internet]. 2013;144: 617–627. Available from: https://hplab-ku.github.io/pdfs/papers/2013_lee_cho.pdf doi: 10.1016/j.actpsy.2013.09.013 24184348

[55] An JY, Seo ER, Lim KH, Shin JH, Kim JB. Standardization of the Korean version of screening tool for depression (Patient Health Questionnaire-9, PHQ-9). Journal of the Korean Society of Biological Therapies in Psychiatry. 2013;19: 47–56.

[56] Lim CE, Cho YS. Response mode modulates the congruency sequence effect in spatial conflict tasks: evidence from aimed-movement responses. Psychol Res [Internet]. 2021;85(5):2047–68. Available from: doi: 10.1007/s00426-020-01376-3 32592067

[57] Egner T, Ely S, Grinband J. Going, going, gone: characterizing the time-course of congruency sequence effects. Frontiers in psychology [Internet]. 2010;1, 154. Available from: https://www.frontiersin.org/articles/10.3389/fpsyg.2010.00154/full 21833220 10.3389/fpsyg.2010.00154 PMC3153769

[58] Ekman P. An argument for basic emotions. Cogn Emot [Internet]. 1992;6(3–4):169–200. Available from: 10.1080/02699939208411068

[59] Russell JA. A circumplex model of affect. J Pers Soc Psychol [Internet]. 1980;39(6):1161–78. Available from: 10.1037/h0077714

[60] Aviezer H, Hassin RR, Ryan J, Grady C, Susskind J, Anderson A, et al. Angry, disgusted, or afraid? Studies on the malleability of emotion perception: Studies on the malleability of emotion perception. Psychol Sci [Internet]. 2008;19(7):724–32. Available from: 10.1111/j.1467-9280.2008.02148.x 18727789

[61] Lee HJ, Cho YS. The effect of threatening facial expressions on inhibition-induced forgetting depends on their task-relevance. Cogn Emot [Internet]. 2020;34(3):526–38. Available from: doi: 10.1080/02699931.2019.1650721 31370745

[62] Gyurkovics M, Stafford T, Levita L. Cognitive control across adolescence: Dynamic adjustments and mind-wandering. Journal of Experimental Psychology: General [Internet]. 2020; 149(6), 1017–1031. Available from: https://eprints.whiterose.ac.uk/152525/3/CogControlManuscript_Revision_Round2_ChangesAccepted.pdf doi: 10.1037/xge0000698 31599622

[63] Correa Á, Rao A, Nobre AC. Anticipating conflict facilitates controlled stimulus-response selection. Journal of Cognitive Neuroscience [Internet]. 2009; 21, 1461–1472. Available from: https://direct.mit.edu/jocn/article/21/8/1461/4720 doi: 10.1162/jocn.2009.21136 18823248 PMC4152723

[64] Bugg JM, Crump MJ. In support of a distinction between voluntary and stimulus-driven control: A review of the literature on proportion congruent effects. Frontiers in psychology [Internet]. 2012; 3, 367. Available from: https://www.frontiersin.org/articles/10.3389/fpsyg.2012.00367 23060836 10.3389/fpsyg.2012.00367 PMC3459019

[65] Ebersole CR, Atherton OE, Belanger AL, Skulborstad HM, Allen JM, Banks JB, et al. Many Labs 3: Evaluating participant pool quality across the academic semester via replication. J Exp Soc Psychol [Internet]. 2016;67:68–82. Available from: 10.1016/j.jesp.2015.10.012

[66] Clayson PE, Larson MJ. Psychometric properties of conflict monitoring and conflict adaptation indices: response time and conflict N2 event-related potentials: Conflict adaptation psychometrics. Psychophysiology [Internet]. 2013;50(12):1209–19. Available from: 10.1111/psyp.12138 23992600

[67] Feldman JL, Freitas AL. An investigation of the reliability and self-regulatory correlates of conflict adaptation. Exp Psychol [Internet]. 2016;63(4):237–47. Available from: doi: 10.1027/1618-3169/a000328 27750519

[68] Allan JL, Johnston M, Campbell N. Unintentional eating. What determines goal-incongruent chocolate consumption? Appetite [Internet]. 2010;54(2):422–5. Available from: doi: 10.1016/j.appet.2010.01.009 20100530

[69] Saunders B, Milyavskaya M, Etz A, Randles D, Inzlicht M. Reported Self-control is not Meaningfully Associated with Inhibition-related Executive Function: A Bayesian Analysis. Collabra Psychol [Internet]. 2018;4(1):39. Available from: 10.1525/collabra.134

[70] Draheim C, Mashburn CA, Martin JD, Engle RW. Reaction time in differential and developmental research: A review and commentary on the problems and alternatives. Psychol Bull [Internet]. 2019;145(5):508–35. Available from: doi: 10.1037/bul0000192 30896187

[71] Hedge C, Powell G, Sumner P. The reliability paradox: Why robust cognitive tasks do not produce reliable individual differences. Behav Res Methods [Internet]. 2018;50(3):1166–86. Available from: doi: 10.3758/s13428-017-0935-1 28726177 PMC5990556

[72] Whitehead PS, Brewer GA, Blais C. Are cognitive control processes reliable? J Exp Psychol Learn Mem Cogn [Internet]. 2019;45(5):765–78. Available from: doi: 10.1037/xlm0000632 30047768

[73] Baker DH, Vilidaite G, Lygo FA, Smith AK, Flack TR, Gouws AD, et al. Power contours: Optimising sample size and precision in experimental psychology and human neuroscience. Psychological Methods [Internet]. 2021;26(3):295–314. Available from: doi: 10.1037/met0000337 32673043 PMC8329985

[74] Rouder JN, Haaf JM. A psychometrics of individual differences in experimental tasks. Psychonomic bulletin & review [Internet]. 2019;26(2), 452–467. Available from: https://link.springer.com/article/10.3758/s13423-018-1558-y 30911907 10.3758/s13423-018-1558-y

[75] Haines N, Kvam PD, Irving LH, Smith C, Beauchaine TP, Pitt MA et al. Learning from the reliability paradox: How theoretically informed generative models can advance the social, behavioral, and brain sciences. PsyArXiv. 2020. Available from: https://www.researchgate.net/profile/Nathaniel-Haines/publication/343844697_Learning_from_the_reliability_paradox_How_theoretically_informed_generative_models_can_advance_the_social_behavioral_and_brain_sciences/links/5f451739a6fdcccc43fe9470/Learning-from-the-reliability-paradox-How-theoretically-informed-generative-models-can-advance-the-social-behavioral-and-brain-sciences.pdf

[76] Hawton K, van Heeringen K, editors. The international handbook of suicide and attempted suicide. Chichester, England: John Wiley & Sons; 2008.

[77] Gazzaley A, D’Esposito M. Top-down modulation and normal aging. Ann N Y Acad Sci [Internet]. 2007;1097(1):67–83. Available from: doi: 10.1196/annals.1379.010 17413013

[78] Kim M-D, Hong S-C, Lee S-Y, Kwak Y-S, Lee C-I, Hwang S-W, et al. Suicide risk in relation to social class: a national register-based study of adult suicides in Korea, 1999–2001. Int J Soc Psychiatry [Internet]. 2006;52(2):138–51. Available from: doi: 10.1177/0020764006061254 16615246

[79] Kim Y, Kim H, Kim D-S. Association between daily environmental temperature and suicide mortality in Korea (2001–2005). Psychiatry Res [Internet]. 2011;186(2–3):390–6. Available from: doi: 10.1016/j.psychres.2010.08.006 20828832

[80] Avila R, Moscoso MAA, Ribeiz S, Arrais J, Jaluul O, Bottino CMC. Influence of education and depressive symptoms on cognitive function in the elderly. Int Psychogeriatr [Internet]. 2009;21(3):560–7. Available from: doi: 10.1017/S1041610209008928 19327202

[81] Noble KG, Korgaonkar MS, Grieve SM, Brickman AM. Higher education is an age-independent predictor of white matter integrity and cognitive control in late adolescence. Dev Sci [Internet]. 2013;16(5):653–64. Available from: doi: 10.1111/desc.12077 24033571 PMC3775010

[82] Van der Elst W, Van Boxtel MPJ, Van Breukelen GJP, Jolles J. The Stroop color-word test: influence of age, sex, and education; and normative data for a large sample across the adult age range: Influence of age, sex, and education; And normative data for a large sample across the adult age range. Assessment [Internet]. 2006;13(1):62–79. Available from: 10.1177/1073191105283427 16443719

[83] Black J, Bond MA, Hawkins R, Black E. Test of a clinical model of poor physical health and suicide: The role of depression, psychosocial stress, interpersonal conflict, and panic. J Affect Disord [Internet]. 2019;257:404–11. Available from: doi: 10.1016/j.jad.2019.05.079 31306991

